# Network meta-analysis of surgical treatment for unstable femoral intertrochanteric fractures

**DOI:** 10.18632/oncotarget.24202

**Published:** 2018-01-02

**Authors:** He-Hui Wang, Wu-Bin Shu, Guan-Hua Lan, Xiao-Bo Zhang, Zhi-Qiang Jiang, De-Hong Xu, Xue-Xun Bao, A-Bing Li

**Affiliations:** ^1^ Department of Orthopedics, Ningbo Yinzhou Second Hospital, Ningbo, Zhejiang, 315100, China

**Keywords:** unstable femoral intertrochanteric fractures, proximal femoral nail antirotation, gamma nail, sliding hip screws, network meta-analysis

## Abstract

In this network meta-analysis, we determined the optimal surgical method for treating unstable femoral intertrochanteric fractures. We searched the EMBASE, Cochrane Library and Medline databases for studies evaluating sliding hip screws (SHS), gamma nail (GN) or proximal femoral nail antirotation (PFNA) methods, and included nine randomized controlled trials that met the inclusion criteria. Our analysis showed no differences in the rates of complications between SHS and PFNA relative to GN (*p* > 0.05). However, the surface under the cumulative ranking curve (SUCRA) score for PFNA (77.6%) was higher than the SUCRA scores for GN (65%) and SHS (7.5%). This suggests PFNA is the better surgical method than GN or SHS for unstable femoral intertrochanteric fractures.

## INTRODUCTION

Femoral intertrochanteric fracture is a common hip fracture that occurs in the elderly [1, 2]. The incidence of femoral intertrochanteric fractures has increased because of an ageing global population [3]. The preferred therapy for femoral intertrochanteric fracture is surgical treatment with rigid fixation, which is associated with early mobilization and fewer complications [4]. The sliding hip screw (SHS) is the gold standard technique for treating stable femoral intertrochanteric fractures, Arbeitsgemeinschaft für Osteosynthesefragen/Orthopedic Trauma Association-31 A1 (AO/OTA 31–A1) [4–6]. However, the optimal treatment method for treating unstable femoral intertrochanteric fractures such as AO/OTA 31-A2 and AO/OTA 31–A3 is controversial. The common treatment methods are proximal femoral nail antirotation (PFNA), gamma nail (GN), and sliding hip screws (SHS). Traditional meta-analyses are inconclusive in determining the best method for treating unstable femoral intertrochanteric fractures because they cannot accurately evaluate 3 or more interventions [7–9]. Network meta-analysis(NMA) is the preferred methodology to compare multiple interventions because it can perform direct and indirect comparisons [10]. We therefore performed a network meta-analysis to determine the optimal treatment method for unstable femoral intertrochanteric fractures based on evaluating the rates of complications between GN, PFNA and SHS.

## MATERIALS AND METHODS

### Literature search

We searched the MEDLINE, EMBASE and the Cochrane Central Register of Controlled Trials (CENTRAL) databases for studies related to treatment of unstable femoral intertrochanteric fractures until May 30, 2017 using search parameters listed in Supplementary List 1. We also manually searched bibliographies of relevant literature to further identify any additional studies that are relevant for our analysis.

### Inclusion and exclusion criteria

Inclusion criteria included (1) randomized controlled clinical trials; (2) studies that reported proximal femoral nail antirotation (PFNA), sliding hip screws (SHS), and gamma nail (GN); (3) patients over 60 years that were diagnosed with unstable peritrochanteric fractures (peritrochanteric or intertrochanteric); and (4) studies that followed up for more than 1 year. Exclusion criteria included (1) duplicate publications; (2) studies with insufficient data; (3) biomechanical, cadaver or model studies; (4) studies on pathological fractures; and (5) non-randomized or retrospective studies, review articles, conference abstracts, letter, or case reports.

### Data extraction

Two researchers (H.H.W. and W.B.S.) independently extracted information such as patient characteristics, first author, publication year, country of origin, follow-up time, treatment methods, and post-operative complications and analyzed the data. Disagreements were resolved by discussion with a third researcher (A.B.L.).

### Risk of bias assessment

The quality of included literatures was assessed by two independent investigators (G.H.L. and X.B.Z.) using the Cochrane risk of bias tool.

### Statistical analysis

We estimated relative risk (RR) with 95% confidence interval (95% CI) for dichotomous variables. Z test was used to measure the pooled effect size [11]. Chi^2^ tests and I^2^ statistic were used to determine heterogeneity in the pooled data. Data with I^2^ > 50% indicated significant heterogeneity [12, 13] and was analyzed by the fixed-effects model, whereas data with I^2^ < 50% was analyzed by the random-effects model. Statistical analysis was performed with the Stata software, version 13.0 (Stata Corporation, College Station, Texas, USA). P < 0.05 was considered statistically significant. Network meta-analysis compares multiple treatments simultaneously by combining direct and indirect evidences of the relative treatment effects [14]. We used inconsistency factors (IF) to estimate heterogeneity in each closed loop and a 95% CI (IF) value of zero indicated absence of statistical significance [15]. Funnel plot analysis was used to estimate small-study effects [16]. We ranked the three interventions for treating unstable femoral intertrochanteric fractures according to Surface Under the Cumulative Ranking Probabilities (SUCRA), which represents the percentage of the area under the curve [17].

## RESULTS

### Baseline characteristics of included studies

**Figure 1:**
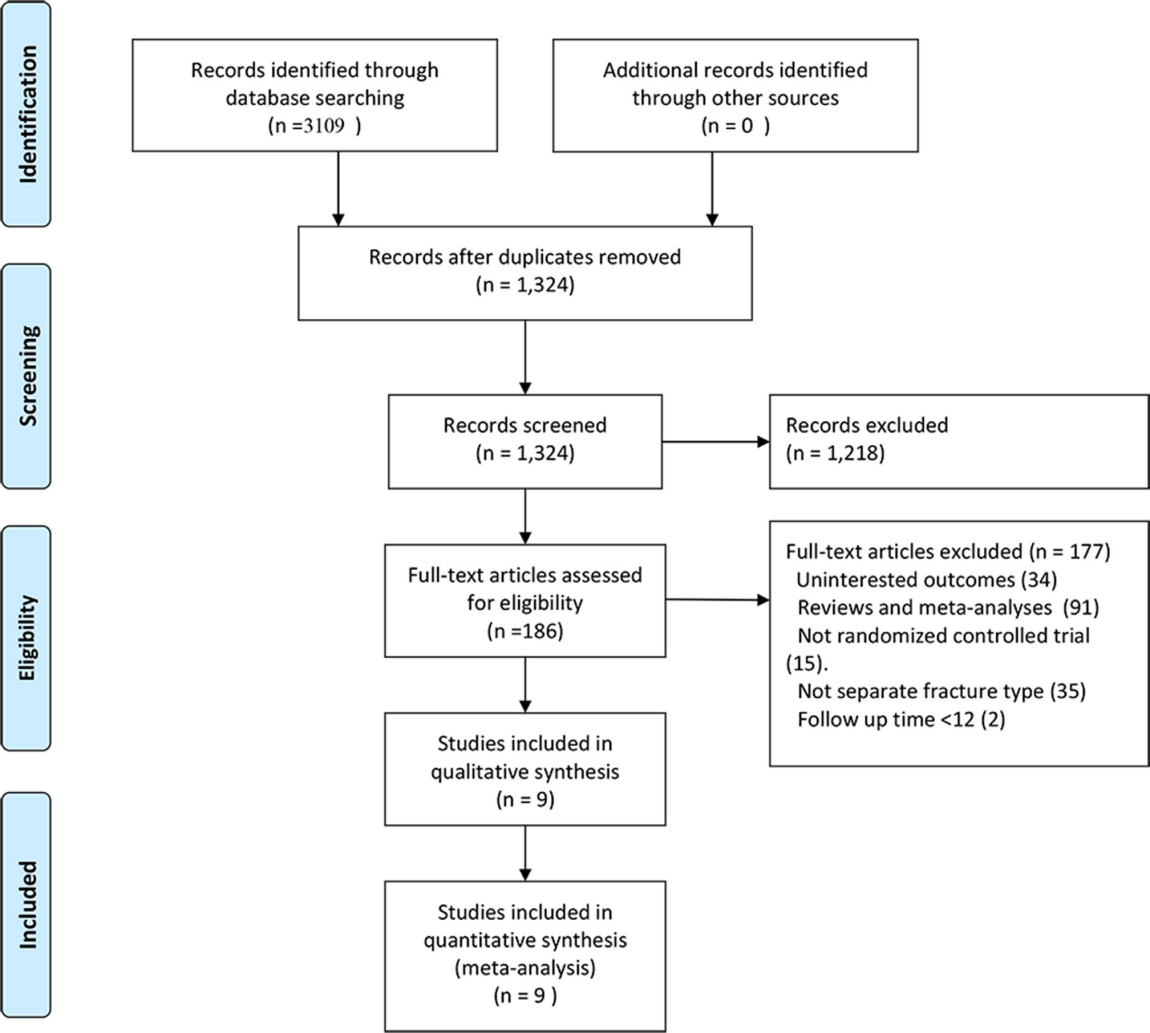
Flow diagram of study selection

**Figure 2:**
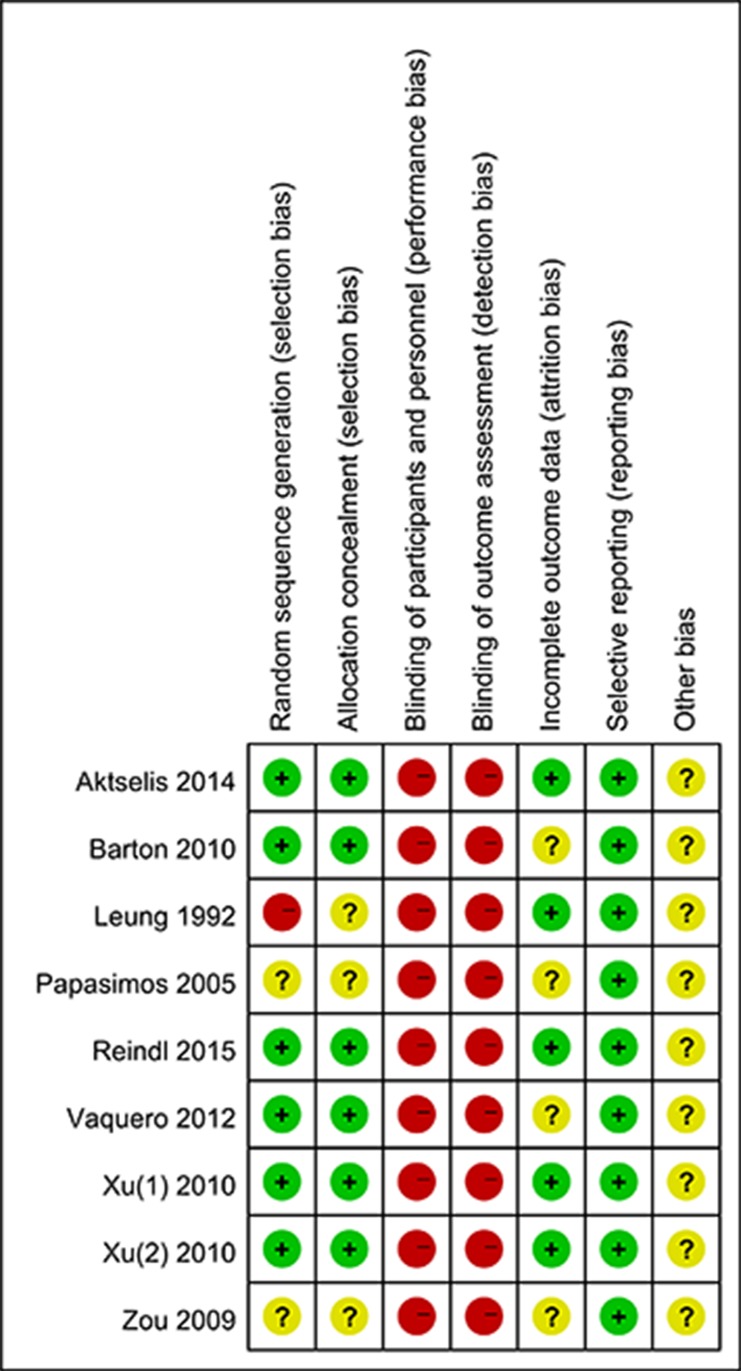
Forest plots show the Cochrane risk of bias assessment of the included studies

We identified 3,109 records by searching MEDLINE, EMBASE and the Cochrane Central Register of Controlled Trials (CENTRAL) databases using search parameters listed in Supplementary List 1. We excluded 1,785 duplicate records and further 1,218 records after reviewing titles and abstracts. After screening the full text of the remaining 186 records, we included 9 articles in our network meta-analysis [2, 5, 18–24]. The study by Zou *et al.* analyzed both unstable and stable fractures [24] and reported the results separately. Therefore, we extracted the data regarding unstable fractures in this study for our analysis. Figure 1 summarizes the selection criteria for enrolling articles in this network meta-analysis. Table 1 summarizes the selected articles in this network meta-analysis. These studies were published between 1992 and 2015 and included 993 participants. Although all of the studies reported randomization, only six trials [2, 5, 18–21] mentioned an adequate randomization technique, and six trials [2, 5, 18–21] reported information of allocation concealment. Due to the nature of the surgical interventions, the blinding was impossible. Figure 2 shows the Cochrane risk of bias assessment of the included studies.

**Table 1:** Characteristics of the included studies

study	Design	Country	Intervention	Age(year)		Number of patients		Follow-up term(month)
			I E	I	E	I	E	
Barton 2010	RCT	UK	GN VS SHS	83.1 (9.5)	83.3 (6.8)	100	110	12
Leung 1992	RCT	HongKong	GN VS SHS	80.8 (8.4)	78.3 (9.5)	63	73	12
Papasimos 2005	RCT	Greece	GN VS SHS	82.8 (NR)	81.4 (NR)	80	40	12
Aktselis 2014	RCT	Greece	GN VS SHS	82.9 (5.8)	83.1 (6.5)	40	40	12
Reindl 2015	RCT	Canadian	GN VS SHS	82.0 (8.6)	80 (9.9)	22	92	12
Zou 2009	RCT	China	PFNA VS SHS	65.0 (13.5)	65 (13.7)	16	11	12
Xu (2) 2010	RCT	China	PFNA VS SHS	78.5 (8.0)	77.9 (7.8)	51	55	12
Vaquero, 2012	RCT	Spain	GN VS PFNA	83.5 (7.4)	83.6 (7.5)	31	33	12
Xu (1) 2010	RCT	China	GN VS PFNA	75.4 (1.0)	76.0 (1.2)	70	66	17

RCT = Randomized Clinical Trial, GN = Gamma nail, PFNA = Proximal femoral nail antirotation, SHS = Sliding hip screws, NR = Not reported.

### Evidence network

**Figure 3:**
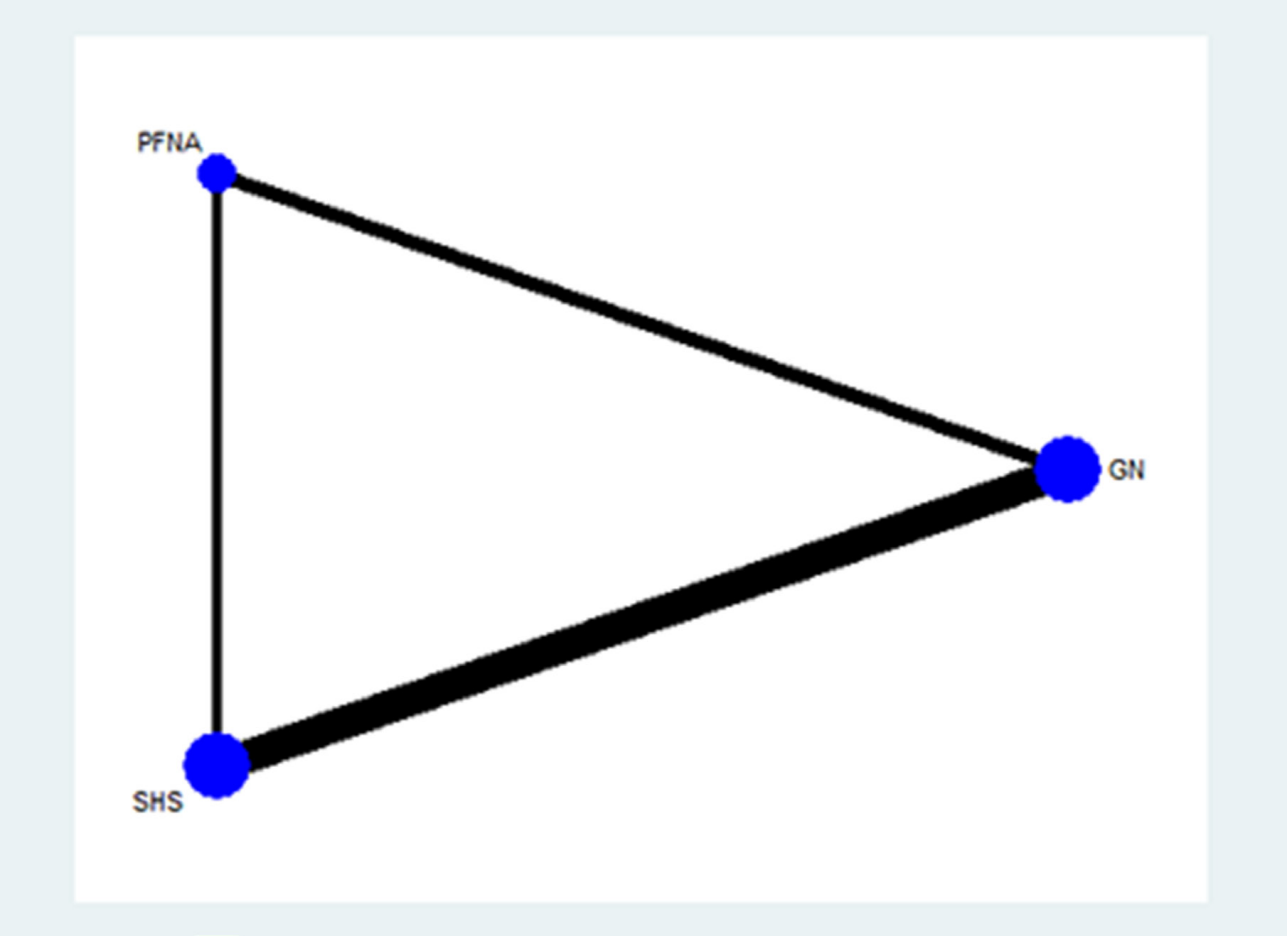
Evidence network of the randomized controlled trials (RCTs) in the network meta-analysis

As shown in Figure 3, the lines in the evidence network represent direct comparison between two directly connected interventions. Interventions without connection are compared indirectly through the network meta-analysis. The width of the lines represents the number of trials, whereas the size of the nodes indicates the overall sample size of GN, PFNA and SHS.

### Contribution plot of network meta-analysis

**Figure 4:**
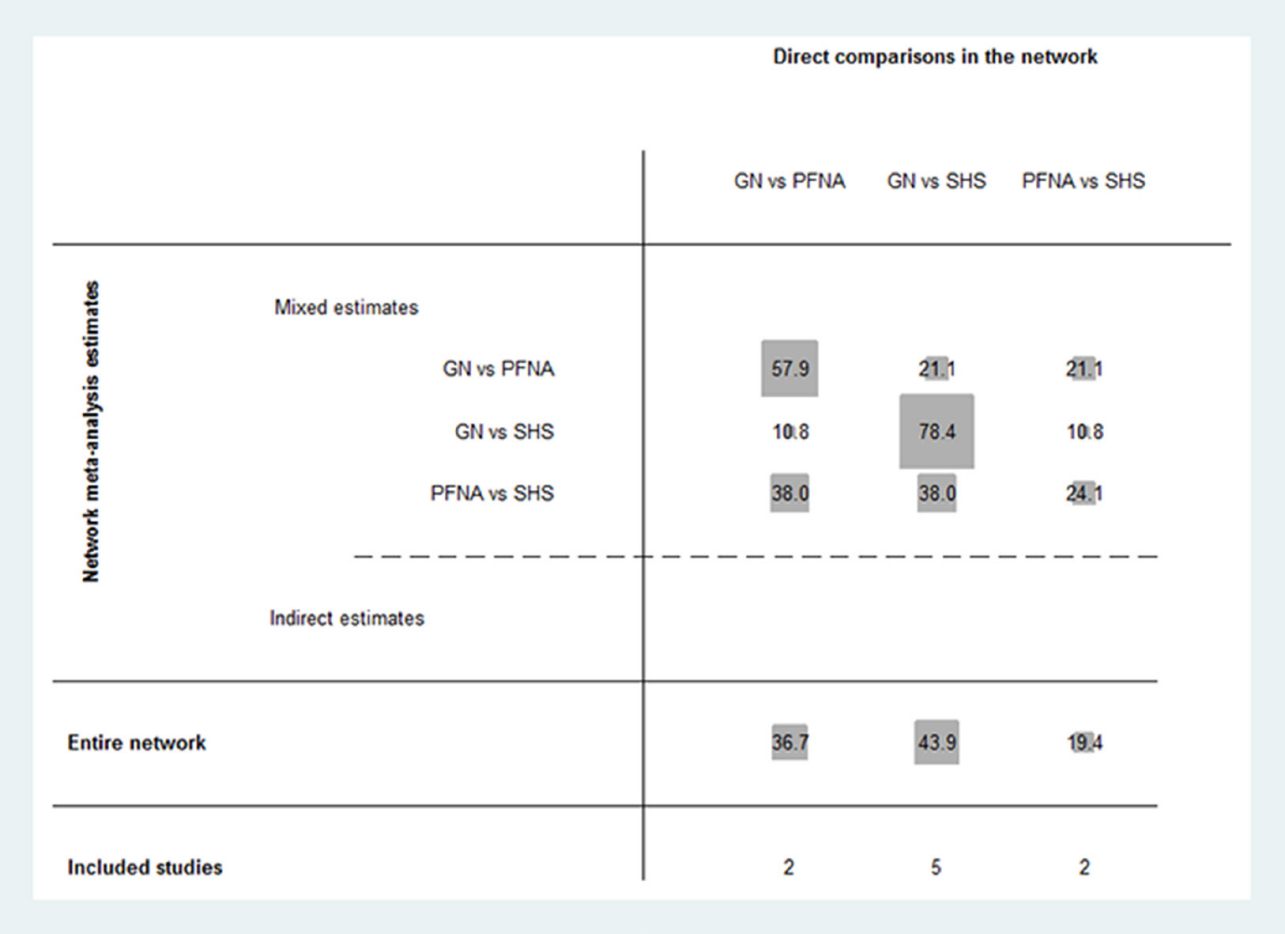
Contribution plot of enrolled studies in the network meta-analysis

Figure 4 shows the contribution of each direct comparison to the network summary effects. Two studies directly compared GN and PFNA [19, 21]. Their percentage contribution for GN vs. PFNA, GN vs. SHS and PFNA vs. SHS was 57.9%, 10.8% and 38.0%, respectively. Their contribution for the total network meta-analysis was 36.7%. Five studies directly compared GN and SHS [2, 5, 18, 22, 23]. Their percentage contribution for GN vs. PFNA, GN vs. SHS and PFNA vs. SHS was 21.1%, 78.4% and 38%, respectively. Their contribution for the total network meta-analysis was 43.9%. Two studies directly compared PFNA and SHS [20, 24]. Their percentage contribution for GN vs. PFNA, GN vs. SHS and PFNA vs. SHS was 21.1%, 10.8% and 24.1%, respectively. Their contribution for the total network meta-analysis was 19.4%.

### Evaluating and presenting assumptions of the network meta-analysis

**Figure 5:**
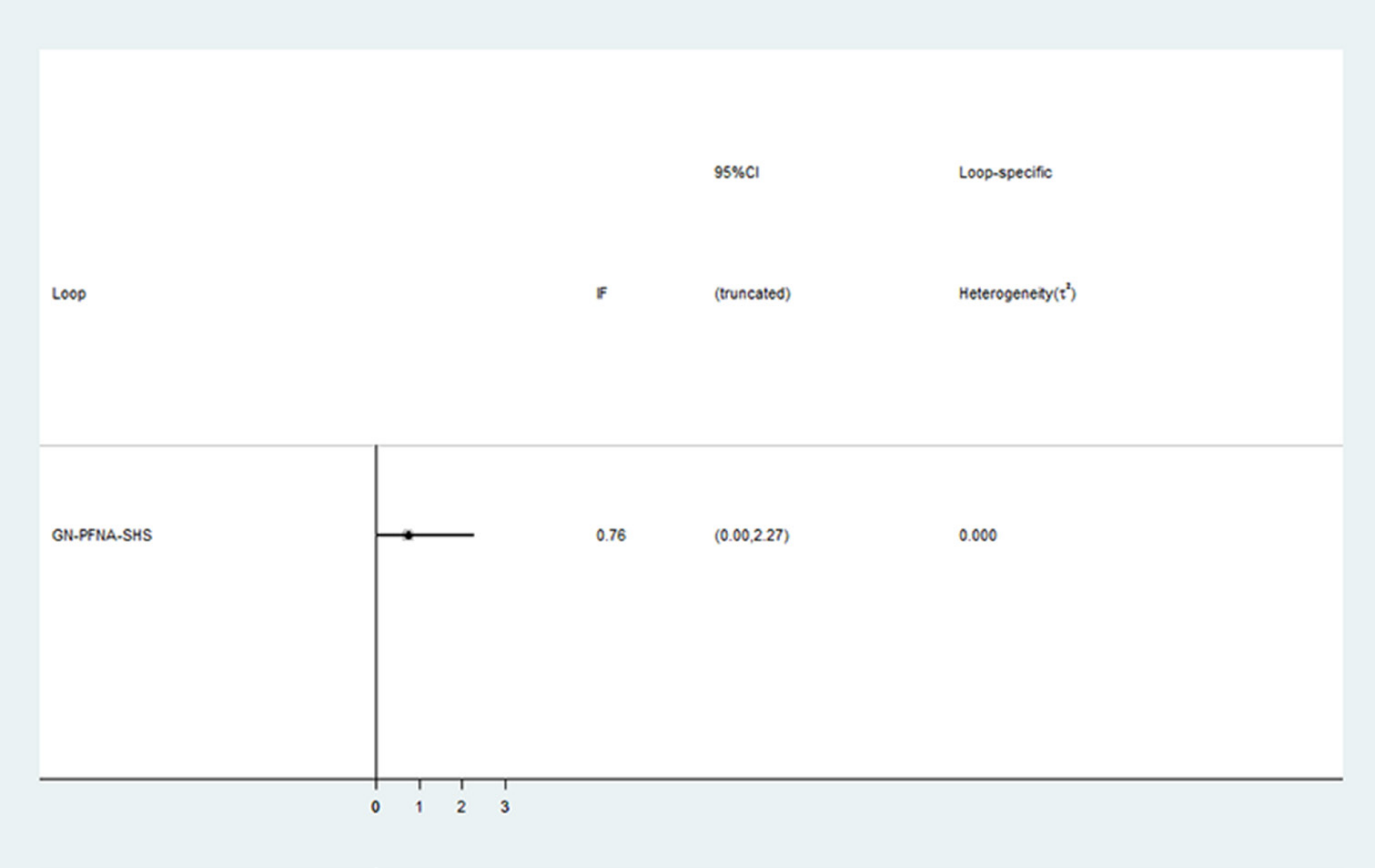
Inconsistency test for direct and indirect comparisons of the enrolled studies in the network meta-analysis

**Figure 6:**
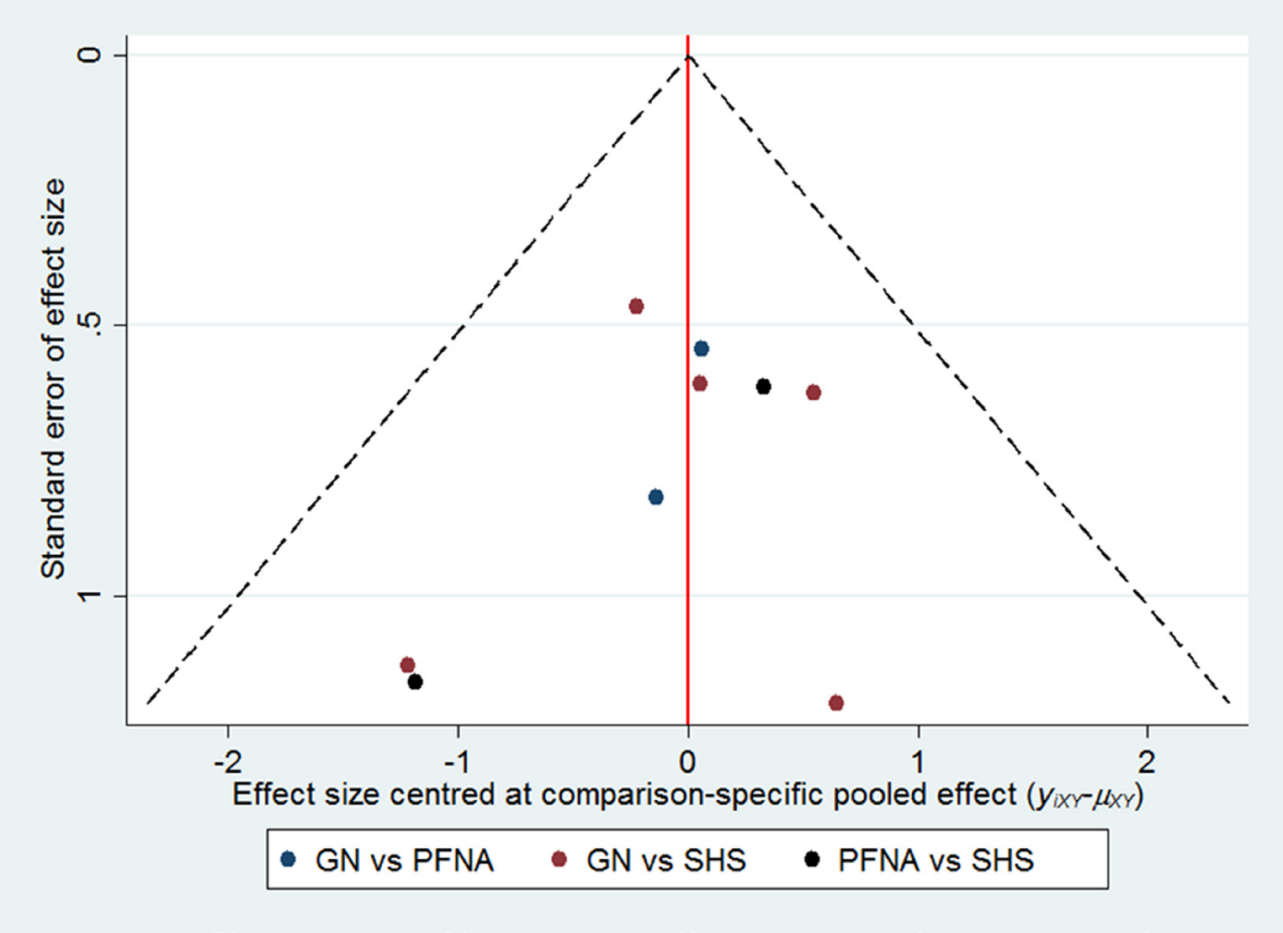
Funnel plots show assessment of publication bias of all the enrolled studies

Figure 5 shows the inconsistency plot used to evaluate the heterogeneity among studies in the closed loop of the network meta-analysis. It was composed of a single triangular GN - PFNA- SHS loop with a 95% CI (IF) value of zero, which demonstrates that our network analysis data was consistent. Furthermore, all the *P* values were greater than 0.05, which indicates that the indirect and direct comparisons of the 3 internal fixations were consistent. As shown in Figure 6, the funnel plot was symmetrical suggesting absence of publication bias.

### Comparison of complication rates

**Figure 7:**
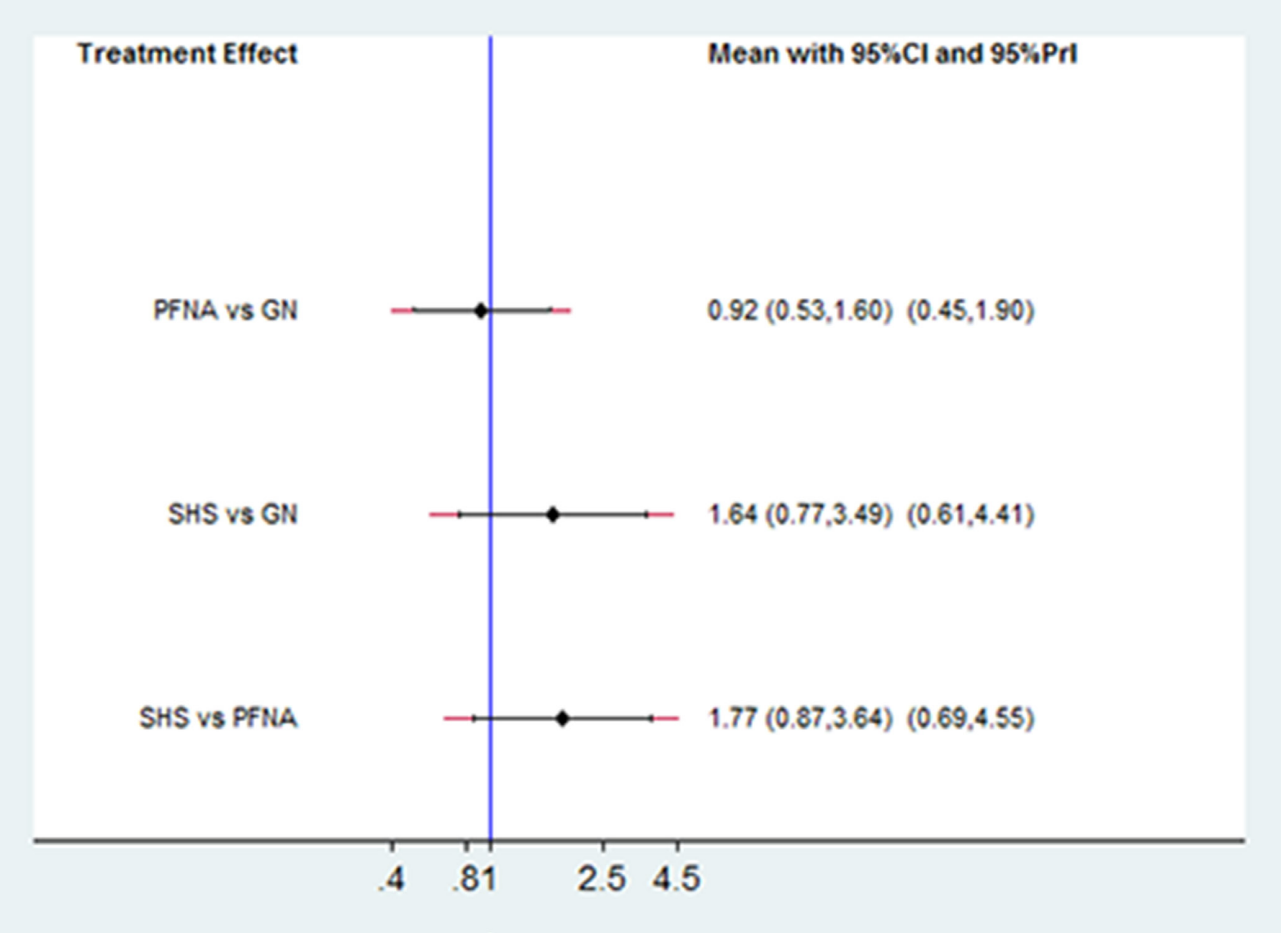
Comparison of the number of complications in the treatment methods for unstable femoral intertrochanteric fractures

**Figure 8:**
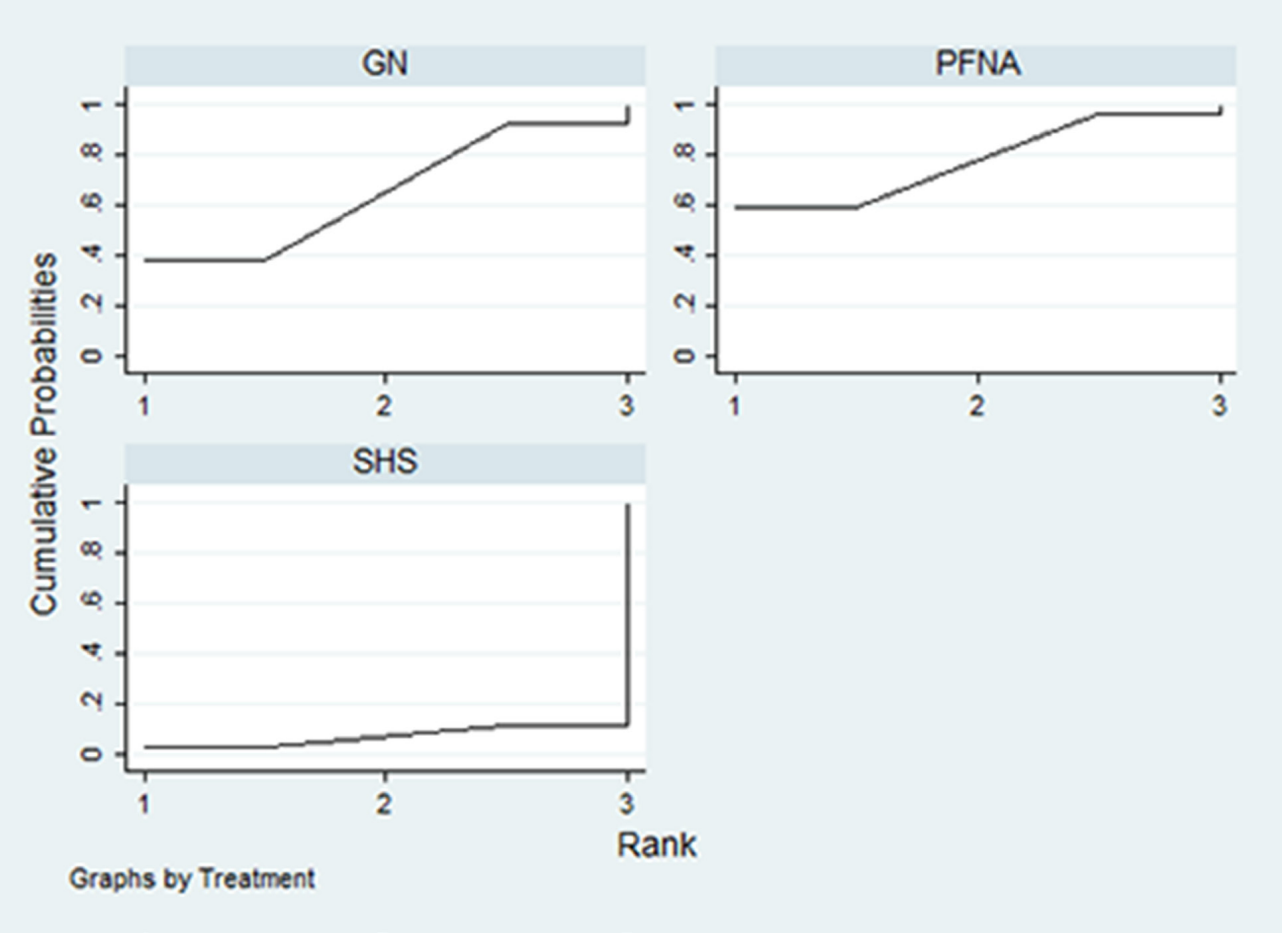
SUCRA probabilities of the three treatment methods for unstable femoral intertrochanteric fractures

The network meta-analysis results showed no significant differences in the complication rates of PFNA and SHS relative to GN for treating unstable femoral intertrochanteric fractures (PFNA: RR = 0.92; 95% CI, 0.53–1.60; *P* = 0.780; SHS: RR = 1.64; 95% CI, 0.77–3.49; *P* = 0.198; Figure 7). The SUCRA scores were 65% for GN, 77.6% for PFNA and 7.5% for SHS (Figure 8). This suggested that PFNA was the optimal treatment for unstable femoral intertrochanteric fractures because of lower complication rates than in GN and SHS.

## DISCUSSION

The best available treatment for unstable femoral intertrochanteric fractures is still a topic of debate. Internal fixation devices such as SHS and intramedullary nails have been developed to fix unstable femoral intertrochanteric fractures. Traditional meta-analyses have compared two internal fixation devices for the treatment of femoral intertrochanteric fractures [7–9]. However, traditional meta-analysis is not conclusive in evaluating more than two internal fixation devices.

Our study is the first article that assessed the three available treatments for unstable femoral intertrochanteric fractures using network meta-analysis. Network meta-analysis is used to compare multiple interventions through direct and indirect comparisons. The aim of this network meta-analysis was to rank the three internal fixations, GN, PFNA and SHS, which are used to treat unstable femoral intertrochanteric fractures based on their rate of complications. Our network meta-analysis showed that the rate of complications were similar in GN, PFNA and SHS. However, PFNA showed the highest SUCRA score of 77.6%, which indicated that PFNA was better than GN and SHS in treating unstable femoral intertrochanteric fractures. Interventions with high SUCRA values are ranked higher [17].

SHS and GN are the most common devices to fix femoral intertrochanteric fractures in the last decade [25, 26]. PFNA is a relatively new device that has been used to fix femoral intertrochanteric fractures [25, 27, 28]. Intramedullary nailing (IMN) has become a popular method of stabilizing the proximal femoral fractures in elderly patients because of short incision, less operative time, minimal blood loss and rapid rehabilitation, which minimizes the risk of medical complications [29, 30]. Several studies have shown that IMN is superior to SHS [30–32]. However, Reindl *et al.* reported that there were no differences between the intramedullary and extramedullary internal fixations based on the primary and secondary clinical outcomes [2]. Queally *et al.* reported that there were no differences between IMN and SHS [33]. Our network meta-analysis also demonstrates no differences between SHS and IMN in regard to complications. However, the SUCRA scores were higher for INM, thereby suggesting lower probability of complications for INM methods than for SHS.

The differences between various intramedullary nailing devices with similar biomechanical principles include the requirement for diaphyseal reaming and the use of anti-rotation systems in fixing the femoral neck. GN is characterized by the need for reaming and the use of lag screw, whereas PFNA is an unreamed nail with an anti-rotational helical blade. Simmermacher *et al.* reported that PFNA was an optimal implant for treating unstable femoral trochanteric fractures because it prevented rotation of femoral head [34]. Biomechanical studies revealed that the helical blade system showed more stability than the conventional lag screw in treating unstable femoral intertrochanteric fractures [35, 36]. However, Zhang *et al.* reported that despite these modifications, the outcomes were similar [37]. Vaquero *et al.* reported that the risk of encountering an intraoperative or local postoperative complication was 71% (22/31; 95% CI: 52–86) for the PFNA treatment group, which was similar to the risk encountered by patients undergoing GN [19]. Our network analysis showed that the RR values for PFNA, GN and SHS were similar. However, the SUCRA scores showed that probability of complications were lower for PFNA than in GN and SHS.

Our network meta-analysis had several strengths. First, we determined efficacy estimates of different treatment strategies based on direct and indirect comparisons. Second, we estimated SUCRA and posterior probabilities of outcomes to distinguish the subtle differences between the three different treatments. We determined that PFNA was the best therapeutic method based on the SUCRA scores. Third, a 95% CI (IF) value of zero demonstrated consistency of our network meta-analysis. Finally, we used a broad and extensive search strategy, which minimized publication bias.

However, this network meta-analysis has several limitations. First, although we used an extensive search strategy, only nine studies were eligible for this network meta-analysis. Second, we focused on adverse events such as cut-out, non-union, intra-operative, post-operative fractures, superficial wounds, wound infection, embolism and fixation failure and did not consider other outcomes like functional scores and patient satisfaction because they were not available. Third, the differences in the postoperative x-ray images between GN, PFNA and SHS resulted in “blinding of outcome” assessment being assessed as “high risk” for 8 studies. Therefore, there may have been performance, detection and attribution bias in our results. Finally, the quality of the recruited studies was not high and hence may have included selection bias or other confounding factors. Hence, in the future, well-designed, high quality, large scale RCT studies are necessary.

In summary, our network meta-analysis shows that PFNA is currently the optional treatment for unstable femoral intertrochanteric fractures.

## SUPPLEMENTARY MATERIALS



## Abbreviations

PFNA: Proximal femoral nail antirotation
GN: Gamma nail
SHS: Sliding hip screws
SUCRA: Surface under the cumulative ranking
IMN: Intramedullary nailing
RR: Relative risk
IF: Inconsistency factors
AO/OTA: Arbeitsgemeinschaft für Osteosynthesefragen/Orthopedic Trauma Association
