# Supplementary material for: Network meta-analysis of surgical treatment for unstable femoral intertrochanteric fractures
Source: Oncotarget. 2018 Jan 2;9(35):24168–77. doi: 10.18632/oncotarget.24202 (PMC5963620; doi:10.18632/oncotarget.24202)
Supplement: Supplementary file 1 [file oncotarget-09-24168-s001.pdf]

Network meta-analysis of surgical treatment for unstable femoral intertrochanteric fractures

SUPPLEMENTARY MATERIALS

SEARCH STRATEGY

CENTRAL (Wiley Online Library)

#1 MeSH descriptor Hip Fractures explode all trees #2 ((hip* or femur* or femoral* or trochant* or pertrochant* or intertrochant* or subtrochant* or intracapsular* or extracapsular* or acetabul*) NEAR fracture*): ti,ab,kw #3 (#1 OR #2) #4 MeSH descriptor Bone Screws, this term only #5 MeSH descriptor Fracture Fixation, Internal explode all trees. #6 MeSH descriptor Bone Plates, this term only. #7 MeSH descriptor Bone Nails, this term only. #8 MeSH descriptor Internal Fixators, this term only. #9 (#4 OR #5 OR #6 OR #7 OR #8) #10 (pin* or nail* or screw* or plate* or fix* or rod *):ti,ab,kw. #11 (#9 OR #10) .#12 (#3 AND #11).

MEDLINE (Ovid interface)

1 exp Hip Fractures/2 ((hip$ or femur$ or femoral$ or trochant$ or pertrochant$ or intertrochant$ or subtrochant$ or intracapsular$ or extracapsular$) adj4 fracture$).tw. 3 or/1-2 4 (bone plate$ or fixation plate$ or bone nail$ or bone screw$ or pin$1 or nail$ or screw$1 or plate$1 or fix$ or rod$1).tw. 5 Internal Fixators/ or Bone Screws/ or Fracture Fixation, Internal/ or Bone Plates/ or Bone Nails/ 6 or/4-5 7 and/3, 6 8 Randomized Controlled Trial.pt. 9 Controlled Clinical Trial.pt. 10 randomized.ab. 11 placebo.ab. 12 randomly.ab. 13 trial.ab. 14 groups.ab. 15 or/8-14 16 exp Animals/ not Humans/ 17 15 not16 18 17 and 7.

EMBASE (Ovid interface)

1 exp hip fractures/ 2 (hip fracture$ or intertrochanteric fracture$ or extracapsular fracture$).tw. 3 ((hip or hips or trochant$ or pertrochant$ or intertrochant$ or subtrochant$ or intracapsular$ or extracapsular$) adj4 fracture$).tw. 4 1 or 2 or 3 5 exp internal fixator/ 6 exp bone plate/ 7 exp fracture fixation/ 8 exp bone nail/ 9 exp bone screw/ 10 (internal fixation device$ or internal fixator$ or internal fixation system$ or internal fracture fixation$).tw. 11 (bone plate$ or fixation plate$ or bone nail$ or bone screw$ or pin? or nail? or nailing$ or plate? or rod? or screw?).tw. 12 5 or 6 or 7 or 8 or 9 or 10 or 11 13. Clinical Trial/ 14. Randomized Controlled Trial/ 15. Randomization/ 16. Single Blind Procedure/ 17. Double Blind Procedure/ 18. Crossover Procedure/ 19. Placebo/ 20. Randomi?ed controlled trial$.tw. 21. Rct.tw. 22. Random allocation.tw. 23. Randomly allocated.tw. 24. Allocated randomly.tw. 25. (allocated adj2 random).tw. 26. Single blind$.tw. 27. Double blind$.tw. 28. ((treble or triple) adj blind$).tw. 29. Placebo$.tw. 30. Prospective Study/ 31. or/13-30 32. Case Study/ 33. Case report.tw. 34. Abstract Report/ or Letter/ 35. or/32-34 36. 31 not 35 37. limit 36 to human 38 4 and 12 and 37.
